# Protective roles of cytoplasmic p21^Cip1^ ^/Waf1^ in senolysis and ferroptosis of lung cancer cells

**DOI:** 10.1111/cpr.13326

**Published:** 2022-08-30

**Authors:** Akira Koyanagi, Hitoshi Kotani, Yuichi Iida, Ryosuke Tanino, Irna D. Kartika, Koji Kishimoto, Mamoru Harada

**Affiliations:** ^1^ Department of Immunology, Faculty of Medicine Shimane University Izumo Shimane Japan; ^2^ Department of Thoracic Surgery Tatikawa General Hospital Niigata Japan; ^3^ Division of Medical Oncology & Respiratory Medicine, Department of Internal Medicine, Faculty of Medicine Shimane University Izumo Shimane Japan; ^4^ Department of Clinical Pathology, Faculty of Medicine University of Muslim Indonesia Sulawesi Indonesia

## Abstract

**Objective:**

Therapy‐induced senescent cancer cells increase the expression of the cyclin‐dependent kinase inhibitors p16^Ink4a^ and p21^Cip1/Waf1^. Given that p21 regulates not only the cell cycle but also cell death, we investigated the roles of p21 in cell death using a p16‐negative A549 human lung adenocarcinoma cell line.

**Methods:**

Senescence was induced by doxorubicin (DXR) or pemetrexed (PEM). The protein expression of p21 was examined by immunoblot. Cell death, reactive oxygen species (ROS) and lipid peroxidation were determined by flow cytometry. ABT‐263 and ABT‐737 were used as senolytic drugs. In vivo growth of A549 cells with different levels of p21 and their sensitivity to PEM were examined in xenograft models.

**Results:**

DXR‐induced senescent A549 cells increased the expression of cytoplasmic p21, and the sensitivity to ABT‐263 was augmented in p21‐knockout A549 (A549‐KOp21) cells. A similar senolytic effect was observed when PEM was combined with ABT‐737. PEM alone induced a higher level of non‐apoptotic cell death, ferroptosis, in A549‐KOp21 cells than in A549 cells. Although there was no difference in the level of lipid peroxidation, ROS levels were higher in PEM‐treated A549‐KOp21 cells than in PEM‐treated A549 cells. A loss of p21 increased the sensitivity of A549 cells to PEM both in vitro and in vivo. A clinical database analysis showed that *CDKN1A* ^high^ lung adenocarcinoma patients had a poorer prognosis compared to *CDKN1A* ^low^ patients.

**Conclusion:**

Cytoplasmic p21, which was increased in therapy‐induced senescent lung cancer cells, plays protective roles in senolysis and ferroptosis.

## Graphical abstract

Doxorubicin or pemetrexed could induce senescence in p21‐expressing parental A549 cells and increase the expression of cytoplasmic p21. However, such drug‐induced senescent A549 cells were relatively resistant to apoptosis by senolytic drugs. By contrast, p21‐knockout A549 (A549‐KOp21) cells increased their sensitivity to senolytic drugs. On the other hand, pemetrexed induced a higher level of non‐apoptotic cell death, ferroptosis, in A549‐KOp21 cells than in A549 cells. These findings highlight the protective roles of cytoplasmic p21 against senolysis and ferroptosis in therapy‐induced senescent lung cancer cells.

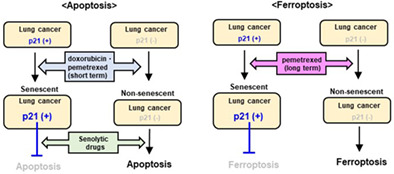

## 1 INTRODUCTION

Cancer cell death is the ultimate goal of anticancer therapies. However, when cancer cells are treated with anticancer drugs, some of them avoid DNA damage by stopping or slowing down their cell cycle, a process known as cellular senescence.^1^ Previous studies suggest that therapy‐induced senescence (TIS) correlates with positive treatment results and could be an index of therapeutic efficacy.^2^, ^3^ However, recent reports have provided conflicting results. Some studies have shown that senescent cancer cells produce several cytokines and factors, known as a senescence‐associated secretory phenotype, which promote recurrence and metastasis of residual cancer cells.^4^, ^5^ TIS cancer cells maintain the potential to resume proliferation for a long period of time.^6^, ^7^, ^8^ Therefore, TIS has been considered a major hurdle that must be overcome to improve the prognosis of cancer patients receiving anticancer therapies.^9^, ^10^

The cyclin‐dependent kinase (CDK) inhibitors p16^Ink4a^ and p21^Cip1/Waf1^ are two key molecules involved in cellular senescence.^8^ TIS is accompanied by increased expression of these CDK inhibitors, and cancer cells attempt to evade cell death by slowing down or stopping their cell cycle because most anticancer drugs mechanistically kill highly proliferative cells. In terms of human adenocarcinoma, inactivation of the *p16* gene is often observed,^11^ suggesting that p21 plays a crucial role in the survival of TIS cancer cells. When anticancer therapies trigger a DNA damage response, p21 expression is enhanced by transcriptional activation of p53.^12^ In addition, it is noteworthy that p21 is involved in various cell functions, such as signalling pathways, regulation of differentiation, cell migration, transcription, DNA repair and apoptosis.^12^ p21 can work as a tumour suppressor as well as a tumour promoter.^13^, ^14^ Despite its role in promoting apoptosis, p21 prevents apoptosis in cancer cells in response to anticancer therapies.^15^, ^16^ Previously, we reported the anti‐apoptotic role of cytoplasmic p21 in CDK4/6 inhibitor‐induced senescent breast cancer cells.^17^

A new approach of selectively removing senescent cells known as senolysis has been proposed. Senolytic drugs can preferentially induce cell death in senescent cancer cells.^18^, ^19^, ^20^, ^21^ ABT‐263 (navitoclax) is one such drug that was identified as an inhibitor against Bcl‐2, Bcl‐xL and Bcl‐w.^22^ In this study, we first examined the senolytic effects of ABT‐263, as well as its analogue ABT‐737,^23^ on drug‐induced senescent human lung adenocarcinoma cells. Senescence was induced by doxorubicin (DXR) or pemetrexed (PEM), which can induce senescence in human breast and lung cancer cells, respectively.^24^, ^25^, ^26^ Second, because we found that PEM alone induced non‐apoptotic cell death in p21‐knockout lung adenocarcinoma cells, we attempted to clarify its mechanism of action. PEM is an anti‐folate drug frequently used for the treatment of nonsquamous non‐small‐cell lung cancer (NSCLC).^27^, ^28^ We found that PEM‐induced cell death was due to ferroptosis, a new type of cell death dependent on iron and lipid peroxidation.^29^, ^30^

## 2 MATERIALS AND METHODS

### 2.1 Cell lines and reagents

Two human lung adenocarcinoma cell lines, A549 and PC9, were obtained from ATCC. A549 has wild‐type p53 and a KRAS mutation, and PC9 has a p53 mutation (R248Q) and an epidermal growth factor receptor mutation. They were maintained in RPMI‐1640 medium (Fujifilm Wako Pure Chemical) supplemented with 10% fetal bovine serum (Invitrogen) and 20 μg/ml gentamicin (Sigma‐Aldrich) and grown at 37°C in a humidified atmosphere with 5% CO_2_. DXR was obtained from Sigma‐Aldrich. ABT‐263 and ABT‐737 were purchased from Active Biochemicals. ABT‐199 was obtained from ChemieTek. A1331852 was purchased from Selleck Chem. PEM was acquired from Eli Lilly. The pan‐caspase inhibitor z‐VAD‐FMK was purchased from Enzo Life Sciences, and both caspase‐8 inhibitor z‐IETD‐FMK and caspase‐9 inhibitor z‐LEHD‐FMK were obtained from R&D Systems. *N*‐acetyl‐l‐cysteine (NAC) was purchased from Nacalai Tesque. Necroptosis‐1, ferroptosis‐1, 3‐methyladenine (3‐MA), and mito‐TEMPO were purchased from Santa Cruz Biotechnology.

### 2.2 Immunoblotting

Immunoblot was performed, as previously reported.^17^ The following primary antibodies were used: anti‐p21^Cip1/Waf1^ (#2947; Cell Signaling Technology), anti‐p16^Ink4a^ (SPC‐1280; StressMarq Biosciences), anti‐p53 (p8999; Sigma‐Aldrich), anti‐phospho‐p53 (#9284; Cell Signaling Technology) anti‐glutathione peroxidase 4 (GPx4) antibody (Cayman), anti‐TATA‐binding protein (TBP; #22006‐I‐AP; Proteintech), anti‐GAPDH (#015‐25473; Fujifilm Wako Pure Chemical) and anti‐β actin (#622102; BioLegend). Nuclear and cytoplasmic proteins were prepared using the LysoPure™ Nuclear and Cytoplasmic Extraction Kit (Fujifilm Wako Pure Chemical). The band intensities were scanned and quantified using the ImageJ software (http://rsb.info.nih.gov/ij/).

### 2.3 Confocal imaging

Confocal imaging was performed, as previously reported.^25^ Fixed cells were stained with SPiDER β‐gal (Dojindo Molecular Technologies) for 30 min. Confocal laser scanning microscopy (FV1000‐D, Olympus) was used.

### 2.4 Flow cytometric analysis

Cell death was assessed using an Annexin V‐FITC Apoptosis Detection Kit (BioVision) and propidium iodide (PI). Analysis was performed using a FACS Calibur flow cytometer (BD Biosciences).

### 2.5 Measurement of reactive oxygen species and lipid peroxidation

Intracellular reactive oxygen species (ROS) were measured using carboxy‐H_2_DCFDA (Molecular Probes). Treated cells were cultured with carboxy‐H_2_DCFDA (10 μM) for 30 min, and the collected cells were analysed by flow cytometry. Lipid peroxidation was measured using Liperfluo (Dojindo Molecular Technologies). Treated cells were cultured with Liperfluo (1 μM) for 30 min, and the collected cells were analysed by flow cytometry.

### 2.6 Cell viability assay

Cell viability was measured using the Cell Counting Kit‐8 (CCK‐8) (Dojindo Molecular Technologies), as previously reported.^17^

### 2.7 Establishment of p21‐knockout, p21‐reexpressing or p21‐overexpressing A549 cell lines

A549 cells were transfected with a p21 CRISPR/Cas9 KO plasmid (#sc‐400013) and p21 HDR plasmid (#sc‐400013‐HDR) (Santa Cruz Biotechnology) using Lipofectamine 3000 (Invitrogen). Thereafter, p21‐knockout A549 (A549‐KOp21) cell colonies were selected using puromycin (Sigma‐Aldrich). To establish p21‐expressing cells from A549‐KOp21 cells, pEB Multi‐Neo vector (Fujifilm Wako Pure Chemical), in which a human *p21* gene was inserted, was transfected into A549‐KOp21 cells using Lipofectamine 3000 (Invitrogen), followed by culture with G418 (Nacalai Tesque). To establish p21‐overexpresing A549 cells, parental A549 cells were similarly transfected with the p21‐encoding pEB Multi‐Neo vector.

### 2.8 In vivo xenograft models

Female BALB nude mice (CLEA) were injected subcutaneously (s.c.) with A549 (2 × 10^6^) cells and Matrigel (BD Biosciences) at a 1:1 volumetric ratio in a 100 μl volume into the right flank. When the tumour diameter was approximately 5–6 mm, the mice were divided into four groups. PEM (100 mg/kg) was intraperitoneally (i.p.) injected on Days 0, 1, 3, 4, 6, 7, 9 and 10 after grouping. On Days 2, 5, 8 and 11 after grouping, cancer‐bearing mice were administered i.p. with ABT‐737 (50 mg/kg). The tumour volume (mm^3^) was calculated as follows: tumour volume = (length × width^2^) ÷ 2. To compare the in vivo growth of A549 cells expressing several levels of p21 and to examine their sensitivity to PEM, female BALB nude mice were similarly s.c. injected with cancer cells. PEM (100 mg/kg) was i.p. injected from Days 8 to 13. All experiments with animals in this study were approved by the Committee on the Ethics of Animal Experiments of the Shimane University Faculty of Medicine (IZ3‐107).

### 2.9 Clinical data analysis

The Kaplan–Meier plotter^31^ was used for univariate analysis of survival time according to *CDKN1A* gene expression in lung cancer patients. The NSCLC database was used for analysis.^32^ Patients were split into low and high expression groups based on the best cut‐off. Outlier array data were excluded for data quality control.

### 2.10 Statistical analysis

To analyse parametric and nonparametric data, Student's *t*‐test (two groups) and analysis of variance (ANOVA) with the Tukey–Kramer test (more than two groups) were used. A *p* value <0.05 was chosen to indicate statistical significance.

## 3 RESULTS

### 3.1 Protective role of p21 in caspase‐dependent senolysis of A549 cells

**FIGURE 1:**
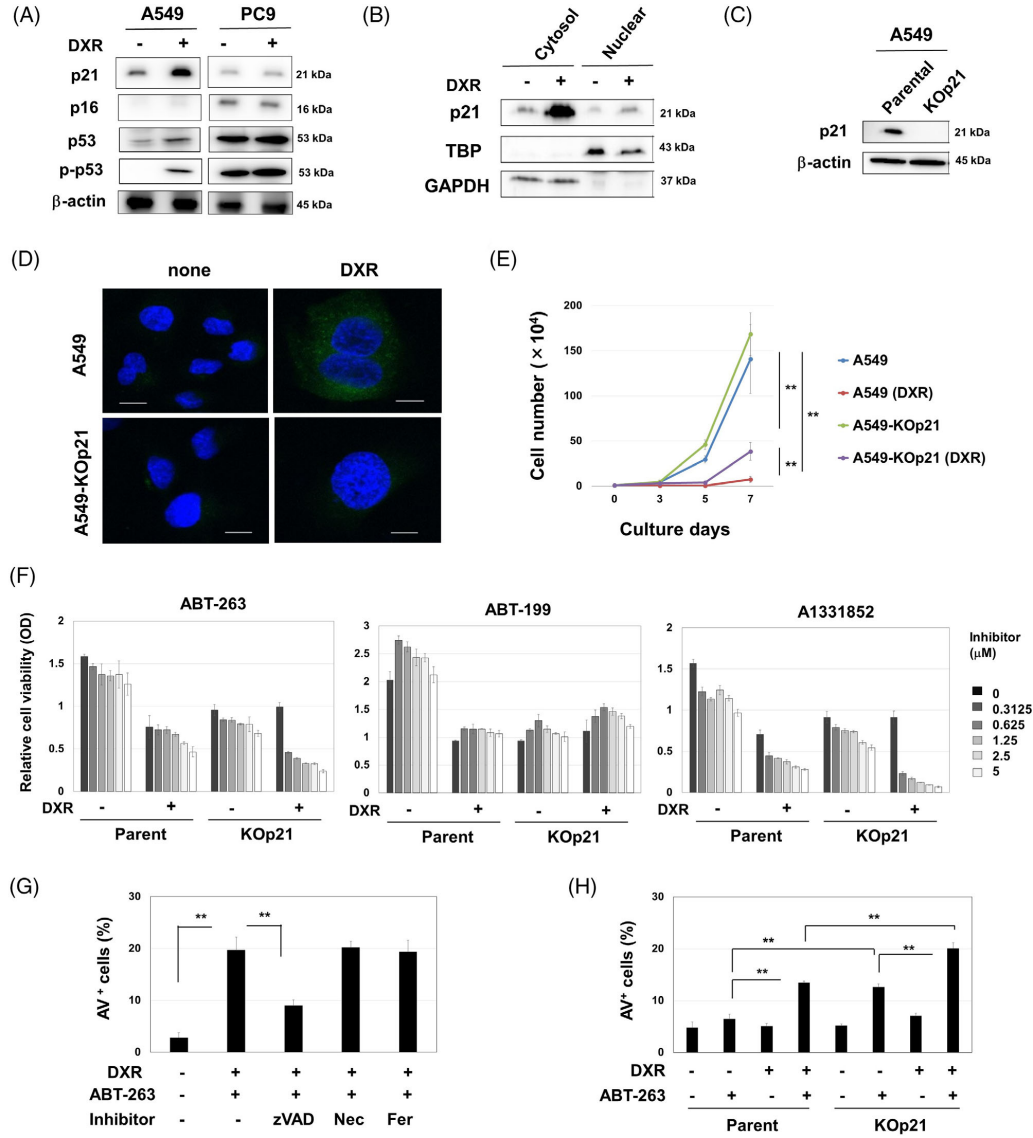
Caspase‐dependent senolysis by ABT‐263 in DXR‐treated A549‐KOp21 cells. (A) Cancer cells were treated with DXR (0.1 μM) for 48 h. Thereafter, immunoblotting was performed. β‐Actin was used as a control. (B) After treating with DXR (0.1 μM) for 48 h, the cytoplasmic and nuclear fractions from A549 cells were separated, and immunoblotting was performed. TBP and GAPDH were used as controls for nuclear and cytoplasmic proteins, respectively. (C) Immunoblot of parental A549 and KO‐p21 cells. (D) Parental A549 and KO‐p21 cells were treated with DXR (0.1 μM) for 48 h and stained with SPiDER β‐gal. Confocal imaging reveals nuclei (blue) and SPiDER β‐gal (green). Scale bar: 10 μm. (E) After treating with DXR (0.1 μM) for 48 h, the growth of A549 and A549‐KOp21 cells was examined in triplicate. **p < 0.01. (F) A549 cells and A549‐KOp21 cells were cultured with or without DXR (0.1 μM) for 48 h. After removing the medium, the cells were cultured with the indicated inhibitors for 48 h. Thereafter, cell viability was determined. Data are the means ± SD of three replicates. (G) After the treatment with DXR (0.1 μM) for 48 h, A549 cells were cultured with ABT‐263 (2.5 μM) with the indicated inhibitors (10 μM) for 48 h. Thereafter, cell apoptosis was measured by flow cytometry. Data are the means ± SD of three replicates. **p < 0.01. (H) Similarly, A549 and A549‐KOp21 cells were cultured with or without DXR (0.1 μM) for 48 h. After harvesting, the cells were cultured with ABT‐263 (2.5 μM) for 48 h, and an apoptosis assay was performed. Data are the means ± SD of three replicates. **p < 0.01. DXR, doxorubicin; Fer, ferrostatin‐1; Nec, necrostatin‐1.

First, we examined the effects of DXR on the expression of p21, p16 and p53 in A549 and PC9 cells. Immunoblots showed that DXR significantly increased p21 expression in A549 cells, but the increase was not apparent in PC9 cells (Figure 1A). P16 was expressed in PC9 cells but absent in A549 cells. DXR apparently increased the expression of p53 and phospho‐p53 in p53 wild‐type A549 cells, whereas p53 mutated PC‐9 cells highly expressed both of them regardless of DXR. DXR treatment remarkedly increased p21 expression in the cytoplasm compared to in the nucleus (Figure 1B). Thereafter, we established A549‐KOp21 cells using the CRISPR/Cas9 method (Figure 1C). Upon DXR treatment, SA β‐Gal was observed in A549 cells but was faint in A549‐KOp21 cells (Figure 1D). DXR treatment stopped the in vitro growth of A549 cells, whereas A549‐KOp21 cells started to grow on Day 5 after DXR treatment (Figure 1E). In terms of cell cycle arrest, there were no significant differences in the absence of DXR treatment, however, suppression of S‐phase subsets was incomplete in DXR‐treated A549‐KOp21 cells (Figure S1A,B).

Next, we examined the effects of ABT‐263, a senolytic drug,^22^ on A549 and A549‐KOp21 cells that were pretreated with DXR (Figure 1F). After 48 h culture with DXR, the cells were cultured with the indicated inhibitors. Without any inhibitors, pretreatment with DXR decreased the subsequent growth of parental A549 cells, whereas this effect was not observed in A549‐KOp21 cells. In terms of A549 cells, pretreatment with DXR increased the sensitivity of A549 cells to ABT‐263; however, this effect was not observed after culture with Blc‐2 inhibitor ABT‐199.^33^ A significant decrease in cell viability was also observed when DXR‐treated A549 cells were cultured with Bcl‐xL inhibitor A1331852. Importantly, senolysis induced by ABT‐263 or A1331852 was augmented in the DXR‐treated A549‐KOp21 cells compared to DXR‐treated A549 cells. In addition, ABT‐263 increased the percentage of Annexin V^+^ cells in DXR‐pretreated parental A549 cells, which was significantly inhibited by pan‐caspase inhibitor z‐VAD, but not by necroptosis inhibitor necrostatin‐1 or ferroptosis inhibitor ferrostatin‐1 (Figure 1G and Figure S1C). Furthermore, the percentage of Annexin V^+^ cells was higher in A549‐KOp21 cells than in parental A549 cells when treated with ABT‐263 alone or with DXR followed by ABT‐263 (Figure 1H and Figure S1D).

### 3.2 Anti‐tumour effects of PEM and/or ABT‐737 on A549 cells both in vitro and in vivo

**FIGURE 2:**
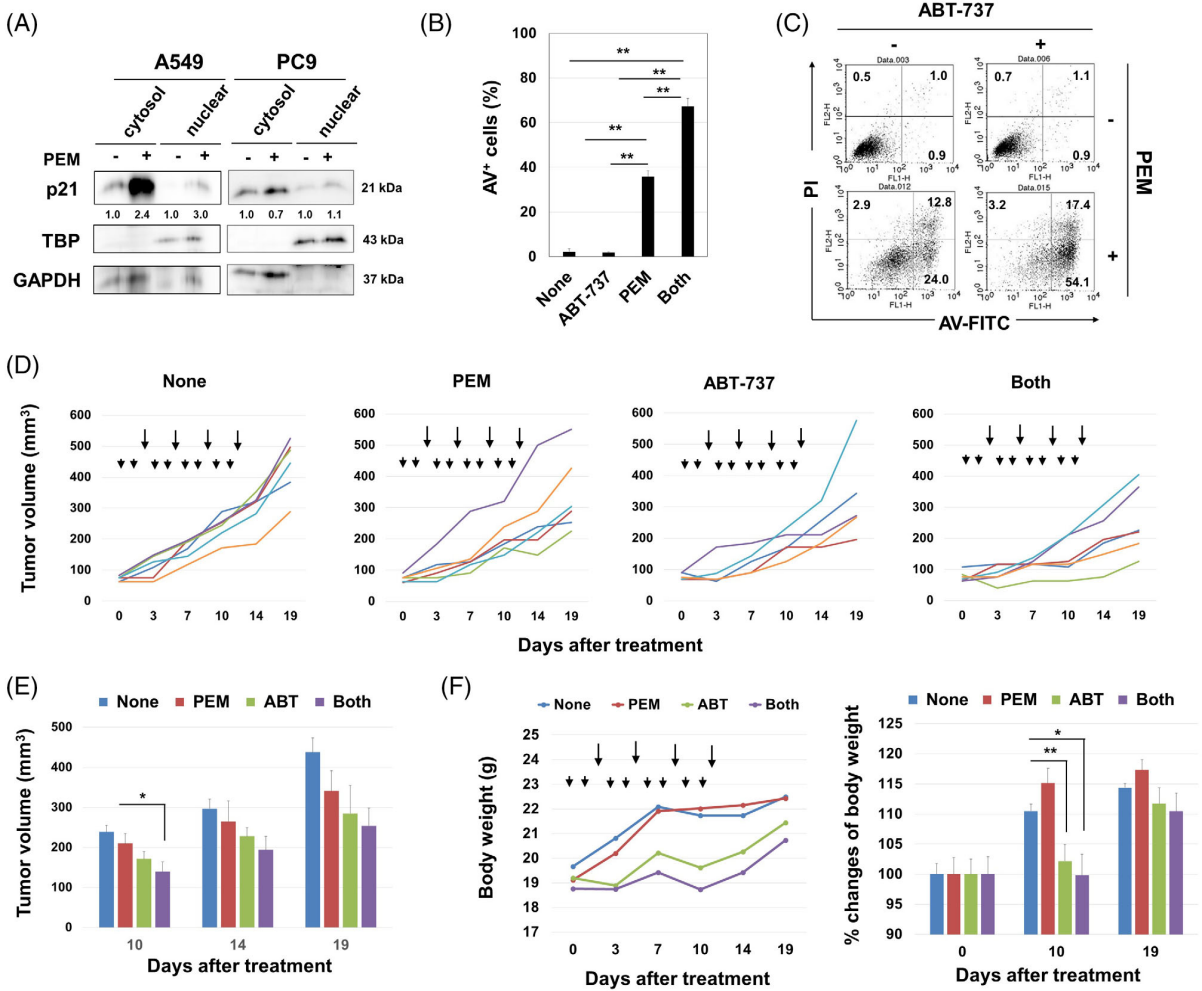
Anti‐tumour effects of PEM and/or ABT‐263 in A549 and A549‐KOp21 cells in vitro and in vivo. (A) Cells were treated with PEM (1 μM) for 48 h. After harvesting, the cytoplasmic and nuclear fractions were separated, and immunoblotting was performed. The band density of cytoplasmic and nuclear p21 was normalized to GAPDH and TBP, respectively. (B) A549 cells were treated with PEM (1 μM) for 48 h. After harvesting, the collected cells were cultured with ABT‐737 (2.5 μM) for an additional 48 h. Thereafter, an apoptosis assay was performed. Data are the means ± SD of three replicates. **p < 0.01. (C) Representative results are shown. The numbers are the percentages of each subset. (D) BALB nude mice were injected with A549 (2 × 10^6^) cells and Matrigel into the right flank. When the tumour diameter was approximately 5–6 mm, PEM (100 mg/kg) (arrowheads) was i.p. injected on Days 0, 1, 3, 4, 6, 7, 9 and 10 after grouping. ABT‐737 (50 mg/kg) (arrows) was i.p. injected on Days 2, 5, 8 and 11 after grouping. Lines represent the growth of individual mice. Each group included 5–6 mice. (E) Tumour sizes are shown. *p < 0.05. (F) Body weights are shown. *p < 0.05, **p < 0.01. PEM, pemetrexed.

PEM is an anti‐metabolite drug that has been frequently used for the treatment of lung adenocarcinoma.^27^, ^28^ In addition, it can induce senescence in A549 cells.^24^, ^25^ In this study, we investigated the role of p21 in PEM‐treated A549 cells as well as PC9 cells. PEM increased the expression of p21 in the cytoplasm and nucleus of A549 cells, but this increase was minimal in PEM‐treated PC9 cells (Figure 2A). A combination of PEM and ABT‐737, the latter of which has senolytic activity similar to ABT‐263,^23^ significantly increased the percentage of Annexin V^+^ A549 cells (Figure 2B,C). In a xenograft model, although monotherapy with PEM or ABT‐737 showed no significant antitumor effects, combining them significantly suppressed the in vivo growth of A549 cells in nude mice on Day 10 compared to the untreated group (Figure 2D,E). However, this difference disappeared after Day 14 probably because variance increased with tumour growth. Although ABT‐737 with or without PEM significantly decreased the body weights of mice, as a general condition of tumour‐bearing hosts, the loss was recovered 9 days after the last treatment with ABT‐737 (Figure 2F).

### 3.3 Protective role of p21 against ferroptosis in PEM‐treated A549 cells

**FIGURE 3:**
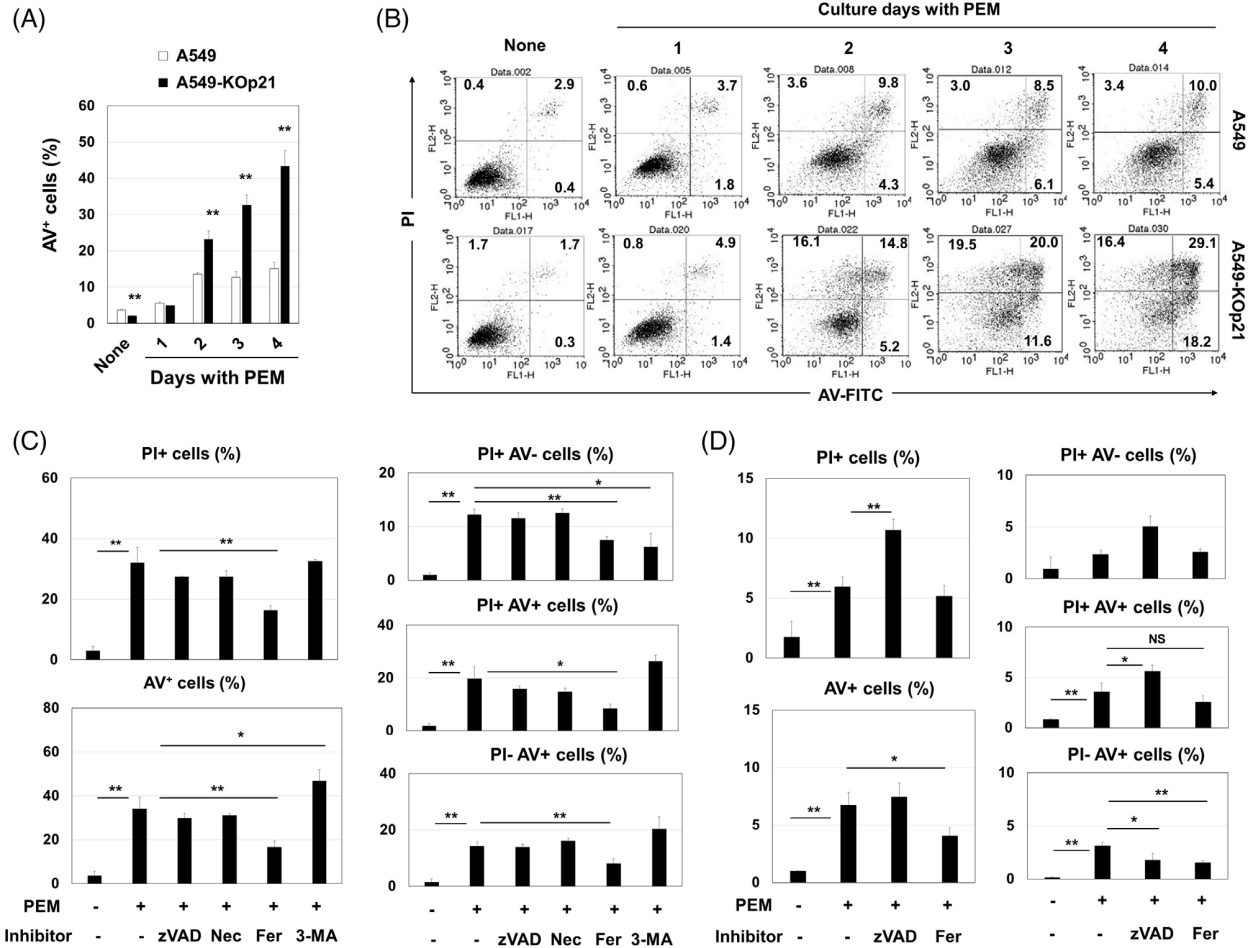
Augmented ferroptosis in PEM‐treated A549‐KOp21 cells. (A) Cells were cultured with PEM (1 μM). On the indicated days, apoptosis assay was done. **p < 0.01. (B) Flow cytometry dot plots are shown. The numbers are the percentages of each subset. (C) A549‐KOp21 cells were cultured with PEM (1 μM) with the indicated inhibitors for 4 days. The bars represent means ± SD of three samples. *p < 0.05, **p < 0.01. (D) Similarly, apoptosis assay was done on parental A549 cells. The bars represent means ± SD of three samples. *p < 0.05, **p < 0.01. NS, not significant; PEM, pemetrexed.

During the in vitro culture of A549‐KOp21 cells with PEM alone, we observed drastic cell death after 4 days of culture. To delineate this observation, we examined kinetic changes in cultured cells. PEM treatment started to increase the percentage of Annexin V^+^ A549 and A549‐KOp21 cells on Day 2 after the initiation of culture, and Annexin V^+^ cells continued to increase thereafter only in the PEM‐treated A549‐KOp21 group (Figure 3A,B). Because we could not establish p21‐knockout PC9 cells, we knocked down p21 in PC9 cells using siRNA transfection. The siRNA p21 (I) decreased the p21 expression in A549 and PC9 cell lines more effectively than siRNA p21 (II) (Figure S2A). PEM treatment for 4 days significantly increased the percentage of Annexin V^+^ p21 siRNA‐transfected PC9 cells, whereas the increase in Annexin V^+^ cells was more apparent in the p21 siRNA‐transfected A549 group (Figure S2B,C).

We further examined what kinds of cell death were induced in PEM‐treated A549‐KOp21 cells. Unexpectedly, adding ferrostatin‐1, a ferroptosis inhibitor, significantly decreased the percentages of both PI^+^ cells and Annexin V^+^ cells, whereas such changes were not observed after adding either zVAD, a pan‐caspase inhibitor, or necrostatin‐1, a necroptosis inhibitor (Figure 3C and Figure S3A). Adding 3‐MA, an autophagy inhibitor, increased the percentage of Annexin V^+^ cells, which suggests a possible protective role of autophagy against the death of PEM‐treated A549‐KOp21 cells. We also tested if ferroptosis could be induced in PEM‐treated parental A549 cells. Although the percentage of PI^+^ PEM‐treated A549 cells was not decreased by zVAD, that of PI^+^ cells increased (Figure 3D and Figure S3B). However, the percentages of Annexin V^+^ cells and PI^−^/Annexin V^+^ cells were decreased by ferrostatin‐1.

### 3.4 Promotion of ferroptosis in PEM‐treated A549‐KOp21 cells

**FIGURE 4:**
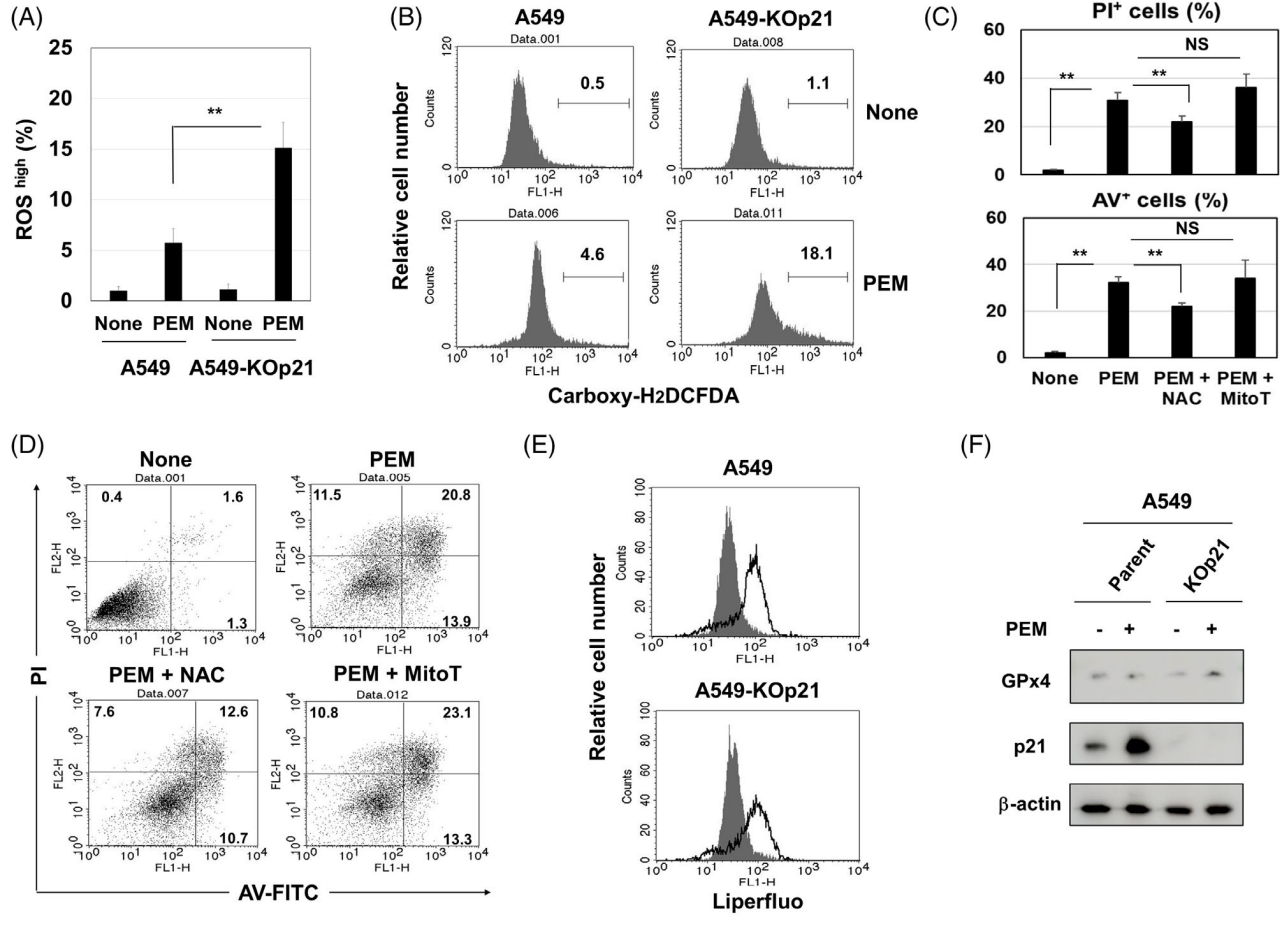
Features of ferroptosis induced in PEM‐treated A549‐KOp21 cells. (A) Cells were cultured with PEM (1 μM). On Day 3, the expression of ROS in cultured cells was examined. **p < 0.01. (B) Flow cytometry results are shown. The numbers are the percentages of ROS^high^ cells. (C) A549‐KOp21 cells were cultured with PEM (1 μM) in the presence of NAC (10 mM) or mitoTEMP (10 μM) for 3 days. Flow cytometric analysis was performed. The bars represent means ± SD of three samples. *p < 0.05, **p < 0.01. NS, not significant. (D) Flow cytometry results are shown. The numbers are the percentages of each subset. (E) A549 and A549‐KOp21 cells were cultured with PEM (1 μM). On Day 3, the expression of lipid peroxidation in cultured cells was examined by flow cytometry. Dark background is a control without PEM. (F) A549 and A549‐KOp21 cells were cultured with PEM (1 μM). On Day 3, the protein expression of GPx4 and p21 was examined by immunoblotting. β‐Actin was used as a control. PEM, pemetrexed; ROS, reactive oxygen species.

Ferroptosis is characterized by increased ROS and lipid peroxidation.^29^, ^30^ In support of this, higher levels of ROS were observed in PEM‐treated A549‐KOp21 cells (Figure 4A,B). In addition, NAC, a ROS scavenger, but not mitoTEMPO, a mitochondria‐targeted antioxidant,^34^ significantly inhibited the induction of PI^+^ and Annexin V^+^ PEM‐treated A549‐KOp21 cells (Figure 4C,D), indicating that mitochondrial ROS were not involved in ferroptosis. Unexpectedly, PEM treatment induced the same level of lipid peroxidation in both A549 and A549‐KOp21 cells (Figure 4E). In addition, the expression of GPx4, a suppressor of lipid peroxidation,^29^ was not decreased by PEM treatment in both A549 and A549‐KOp21 cells (Figure 4F).

### 3.5 Sensitivity of A549 cells expressing different levels of p21 to PEM both in vitro and in vivo

**FIGURE 5:**
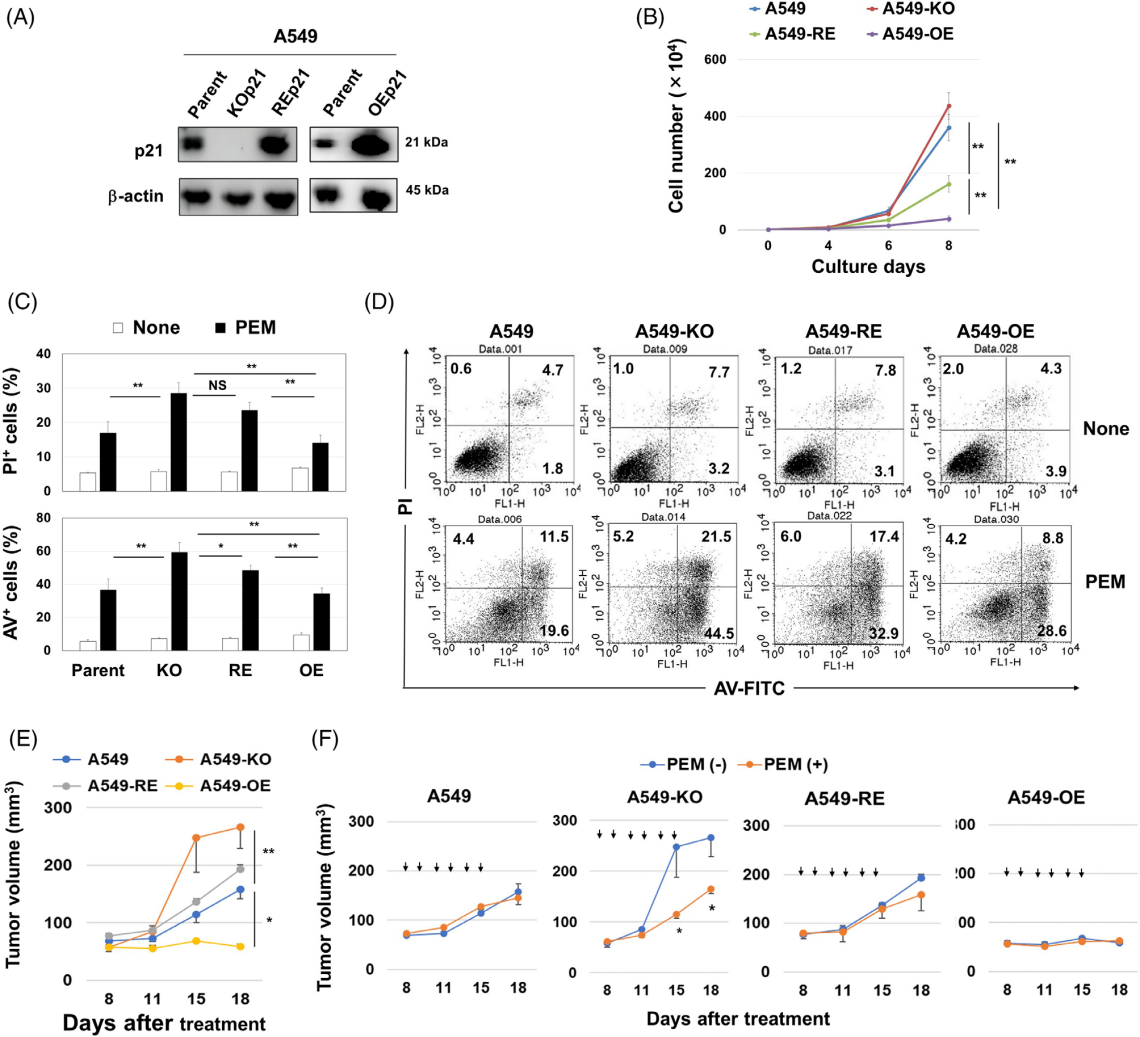
The protective role of p21 in A549 cells towards PEM both in vitro and in vivo. (A) Western blotting analysis was performed on parental A549, A540‐KOp21, A549‐REp21 and A549‐OEp21 cell lines. (B) The growth of four cell lines (1 × 10^4^) was examined. The bars represent means ± SD of three replicates **p < 0.01. (C) After treating with PEM (1 μM) for 4 days, low cytometry was performed. *p < 0.05, **p < 0.01. NS, not significant. (D) Flow cytometry dot plots are shown. The numbers are the percentages of each subset. (E) Female BALB nude mice were injected with four cell lines (2 × 10^6^) with Matrigel into the right flank. Each group included six mice. *p < 0.05, **p < 0.01. (F) Similarly, BALB nude mice were injected with four cell lines. In some mice, PEM (100 mg/kg) (arrows) was i.p. injected from Days 8 to 13. Tumour volume (mm^3^) is shown. Each group included six mice. PEM, pemetrexed

To further investigate the role of p21 in ferroptosis of PEM‐treated A549 cells, we established p21‐reexpressing A549 cells from A549‐KOp21 cells (A549‐REp21) and p21‐overexpressing A549 cells from parental A549 cells (A549‐OEp21) (Figure 5A). The in vitro growth of A549‐KOp21 cells was slightly faster than that of parental A549 cells, but the difference was not significant. By contrast, the growth of A549‐REp21 cells was significantly slower than that of parental A549 cells, and A549‐OEp21 cells grew even more slowly (Figure 5B). Next, cell death in these cell lines was examined after a 4‐day culture with PEM (Figure 5C,D). The percentages of PI^+^ or Annexin V^+^ cells were significantly increased in PEM‐treated A549‐KOp21 cells compared to the other groups, except for the percentages of PI^+^ cells among PEM‐treated A549‐REp21 cells. The in vivo growth of A549‐KOp21 in nude mice was significantly faster than that of parental A549 in untreated nude mice, while that of A549‐OEp21 was significantly slower than the other cell lines (Figure 5E). When these tumour‐bearing mice were treated with PEM, significant growth suppression was observed only in A549‐KOp21‐grafted mice (Figure 5F).

### 3.6 Poor prognosis in lung adenocarcinoma patients with low expression of *CDKN1A*

**FIGURE 6:**
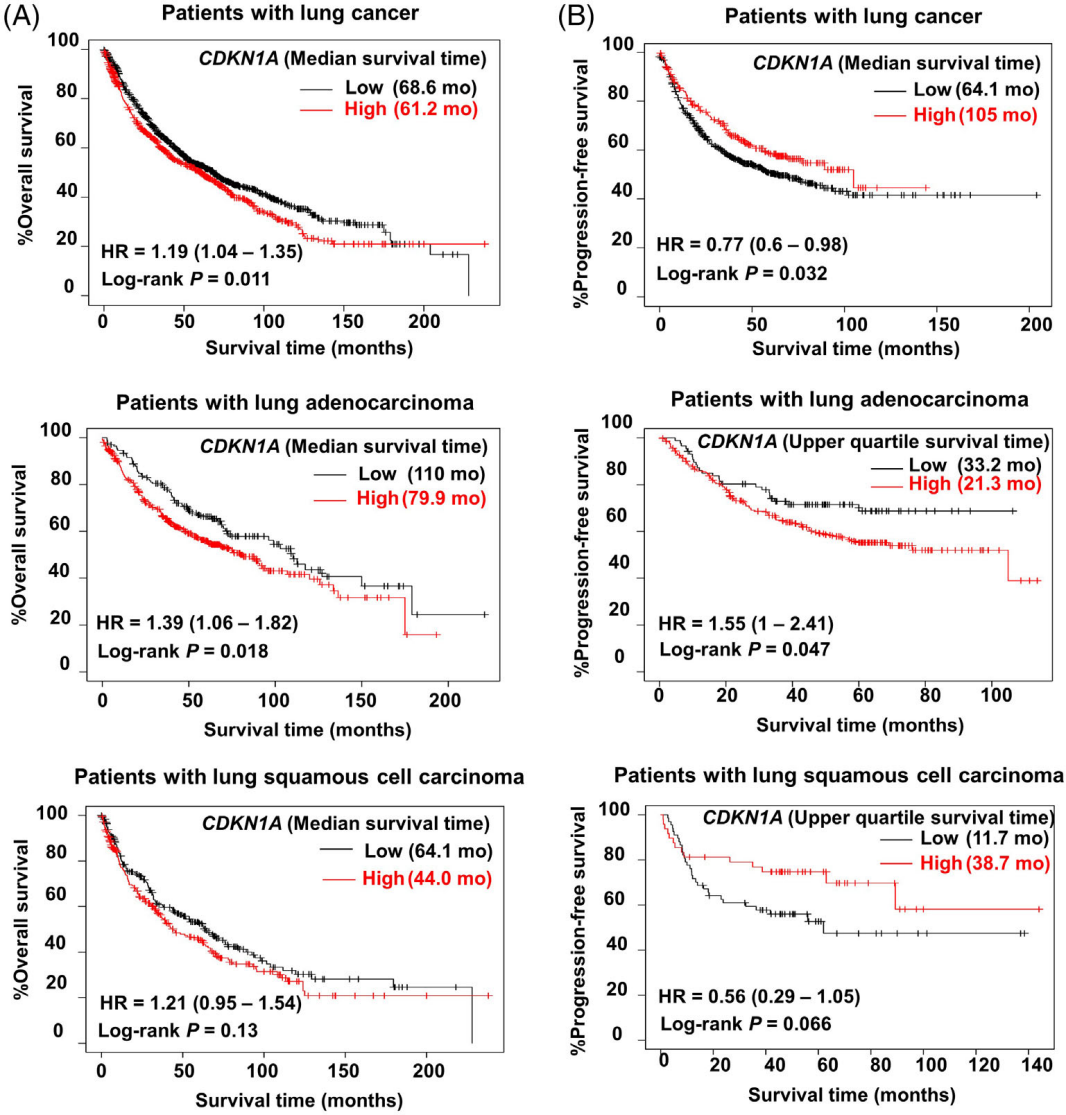
Differences in prognosis according to the level of p21 expression in lung cancer patients. Kaplan–Meier plotter univariate analysis of overall survival time (A) and progression‐free survival time (B) according to *CDKN1A* mRNA expression in lung cancer patients. Version 2021 of the database was used for analysis. Outlier array data were excluded for array quality control. Patients were split into low and high expression groups based on the optimal cut‐off.

We finally investigated whether a difference in *CDKN1A* expression had any influence on the prognosis of lung cancer patients. The Kaplan–Meier plotter was used for univariate analysis of survival time according to *CDKN1A* expression in lung cancer, lung adenocarcinoma, and squamous cell carcinoma patients. Patients were split into low and high expression groups using the best cut‐off. Based on mRNA levels, patients with lung cancer and lung adenocarcinoma with high *CDKN1A* expression showed significantly poorer overall survival compared to those with low *CDKN1A* expression (Figure 6A). However, this was not the case with squamous cell carcinoma patients. Likewise, patients with adenocarcinoma with high *CDKN1A* expression showed significantly poorer progression‐free survival compared to those with low *CDKN1A* expression. However, lung cancer patients or squamous cell carcinoma patients with high *CDKN1A* expression showed better progression‐free survival compared to those with low *CDKN1A* expression (Figure 6B).

## 4 DISCUSSION

Although TIS had been considered an index of tumour‐suppressive phenomena,^35^, ^36^ recent studies suggest an opposite possibility that cell cycle arrest in TIS cancer cells is not always irreversible, and that these cells can potentially re‐enter the cell cycle and grow again.^7^, ^8^ Growth arrest in TIS cancer cells is associated with increased expression of CDK inhibitors, including p16 and p21,^8^ both of which are key regulators in the cell cycle of senescent cancer cells. However, because p16 is frequently lost in NSCLC,^11^ we focused on p21 in this study. Interestingly, p21 plays various roles in tumours depending on its intracellular localization.^37^, ^38^ Several studies have reported that cytoplasmic p21 inhibits apoptosis by inhibiting pro‐caspase‐3, caspase‐8, caspase‐10 and apoptosis signal‐regulating kinase 1.^39^, ^40^ In this study, we showed that DXR treatment induced growth arrest as well as increased expression of p21 in A549 cells, particularly in the cytoplasm. In TIS A549 cells, p21 may play two contrasting roles: regulation of the cell cycle in the nucleus and protection from cell death in the cytoplasm. Namely, p21 in TIS cancer cells can work as a double‐edged sword: a tumour suppressor and a tumour promoter.^14^ However, it has not been clarified how p21 prevented drug‐induced senolysis of A549 cells. Some reports suggest that p21 can inhibit caspase‐3 cleavage via direct binding.^15^, ^16^ However, our trial to co‐precipitate p21 and caspase‐3 was unsuccessful. Nevertheless, zVAD, a pan‐caspase inhibitor, inhibited senolysis (Figure 1G). Therefore, we suppose that caspases other than caspase‐3 could be involved in senolysis. We plan to define the detailed mechanisms in the next study.

ABT‐236 significantly decreased the viability of DXR‐treated A549 cells. ABT‐263 is an inhibitor of Bcl‐2, Bcl‐xL and Bcl‐w,^22^ and senolysis in DXR‐treated A549 cells was induced by Bcl‐xL inhibitor A1331851 (Figure 1F). In addition, senolysis in DXR‐treated A549 cells was mainly caused by caspase‐dependent apoptosis. Given that TIS cells increase cytoplasmic p21 expression, p21 may have inhibitory effects on senolysis. Likewise, we previously reported the protective role of cytoplasmic p21 in the apoptosis of CDK4/6 inhibitor‐induced senescent breast cancer cells.^17^ In this study, we utilized PEM, an anti‐folate drug, which has been widely used for the treatment of NSCLC patients^27^, ^41^ and can induce senescence in NSCLC cell lines, including A549 cells.^24^, ^25^ PEM treatment significantly increased cytoplasmic p21 in A549 cells, and to a lesser degree in PC9 cells. In addition, senolytic drug ABT‐737 preferentially induced cell death in PEM‐treated A549 cells in vitro and synergized with PEM in vivo (Figure 2D,E). These results suggest that targeting TIS cancer cells using senolytic drugs could be a promising strategy to eliminate residual cancer cells after chemotherapy.

In comparing the effects of PEM on A549 and A549‐KOp21 cells, we observed drastic cell death in A549‐KOp21 cells 2 days after the initiation of culture (Figure 3A,B). Additional studies with a panel of inhibitors revealed that this cell death was not induced by apoptosis, but by ferroptosis. Ferroptosis is a new type of cell death that depends upon Fe^2+^ and ROS, particularly lipid peroxidation.^29^, ^30^ We judged that cell death in PEM‐treated A549‐KOp21 cells was due to ferroptosis based on its significant inhibition by ferrostatin‐1, as well as the lack of inhibition by zVAD, nescrostain‐1 or 3‐MA. Although lipid peroxidation was induced in both PEM‐treated parental A549 and A549‐KOp21 cells at a similar level, the level of ROS was higher in PEM‐treated A549‐KOp21 cells than in A549‐KOp21 cells (Figure 4A,B). In the flow cytometric analysis, all percentages of PI^+^/Annexin V^−^, PI^+^/Annexin V^+^ and PI^−^/Annexin V^+^ PEM‐treated A549‐KOp21 cells were decreased by adding ferrostatin‐1. In general, Annexin V^+^ cells are considered apoptotic cells, whereas our results indicate that it is difficult to differentiate ferroptosis from other types of cell death based solely on the Annexin V/PI assay. In addition, the percentages of PI^+^ or Annexin V^+^ cells were significantly inhibited by NAC, a ROS scavenger, but not by mitoTEMPO, a mitochondrial‐targeting ROS inhibitor,^34^ indicating that mitochondrial lipid peroxidation was not involved in PEM‐induced ferroptosis in A549‐KOp21 cells. Furthermore, GPx4 is generally decreased in ferroptotic cells,^42^ whereas there was no decrease in the expression of GPx4 of PEM‐treated A549 and A549‐KOp21 cells (Figure 4F). Intriguingly, a low level of ferroptosis was induced even in PEM‐treated parental A549 cells in which the expression of cytoplasmic p21 was increased (Figure 2A). Based on these results, we speculated that PEM increased lipid peroxidation in both parental A549 and A549‐KOp21 cells, but that increased cytoplasmic p21 in senescent A549 cells hindered the induction of ROS, other than lipid peroxidation. By contrast, such feedback suppression was not elicited in PEM‐treated A549‐KOp21 cells, resulting in promoted ferroptosis. Alternatively, there are several studies examining the role of p21 in ferroptosis of cancer cells. The p53–p21 axis suppresses stress (nutrient deprivation)‐induced ferroptosis in cancer cells,^43^ and p21 can be a barrier to ferroptosis.^44^, ^45^ However, the mechanism behind how lipid peroxidation can induce ferroptosis has not been fully elucidated.^46^

We determined whether *CDKN1A* gene expression influences the prognosis of lung cancer patients using a clinical database.^32^ *CDKN1A* ^high^ lung adenocarcinoma patients showed a poorer prognosis compared to *CDKN1A* ^low^ patients (Figure 6A). Similar results were observed in terms of progression‐free survival of lung adenocarcinoma patients (Figure 6B). However, this trend was not observed in lung squamous cell carcinoma patients, where *CDKN1A* ^high^ patients demonstrated a better prognosis than *CDKN1A* ^low^. However, these data should be carefully interpreted as treatment modalities differ between lung adenocarcinoma and squamous cell carcinoma patients because p21 plays multiple roles in cancer cell biology. In addition, because these database analyses are based on the mRNA expression, it is unclear for the cytoplasmic CDKN1A expression with prognosis. We plan to work on these issues in the future.

In conclusion, the present study demonstrates for the first time that PEM, widely used for maintenance therapy for adenocarcinoma lung cancer patients, can induce ferroptosis; however, increased p21 in PEM‐induced senescent lung cancer cells may downregulate these effects. Given that increased p21 in TIS cancer cells could hinder the induction of apoptosis and ferroptosis, transient inhibition of p21 could be therapeutically beneficial. However, inadequate induction of p21 expression may actually lead to tumour growth.^47^ Given that ferroptosis has received much attention in anticancer therapy,^48^, ^49^ further studies are needed to elucidate the precise mechanism of PEM‐induced ferroptosis.

## AUTHOR CONTRIBUTIONS

Akira Koyanagi and Mamoru Harada designed the experiment. Akira Koyanagi, Hitoshi Kotani, Yuichi Iida, Irna D. Kartika and Mamoru Harada carried out the experiments and generated data. Ryosuke Tanino analysed the public data. Akira Koyanagi, Koji Kishimoto and Mamoru Harada drafted the manuscript.

## CONFLICT OF INTEREST

The authors declare no conflicts of interest.

## Supporting information


**FIGURE S1** Cell cycle of A549 and A549‐KOp21 cells after DXR treatment

**FIGURE S2** Increased sensitivity of siRNA‐mediated knockdown PC9 and A549 cells to PEM

**FIGURE S3** PEM‐induced ferroptosis in A549‐KOp21 and parental A549 cells

## Data Availability

All data are available upon request to the corresponding author.
