# Supplementary figures and images for: Protective roles of cytoplasmic p21Cip1 /Waf1 in senolysis and ferroptosis of lung cancer cells
Source: Cell Prolif. 2022 Aug 30;55(12):e13326. doi: 10.1111/cpr.13326 (PMC9715353; doi:10.1111/cpr.13326)

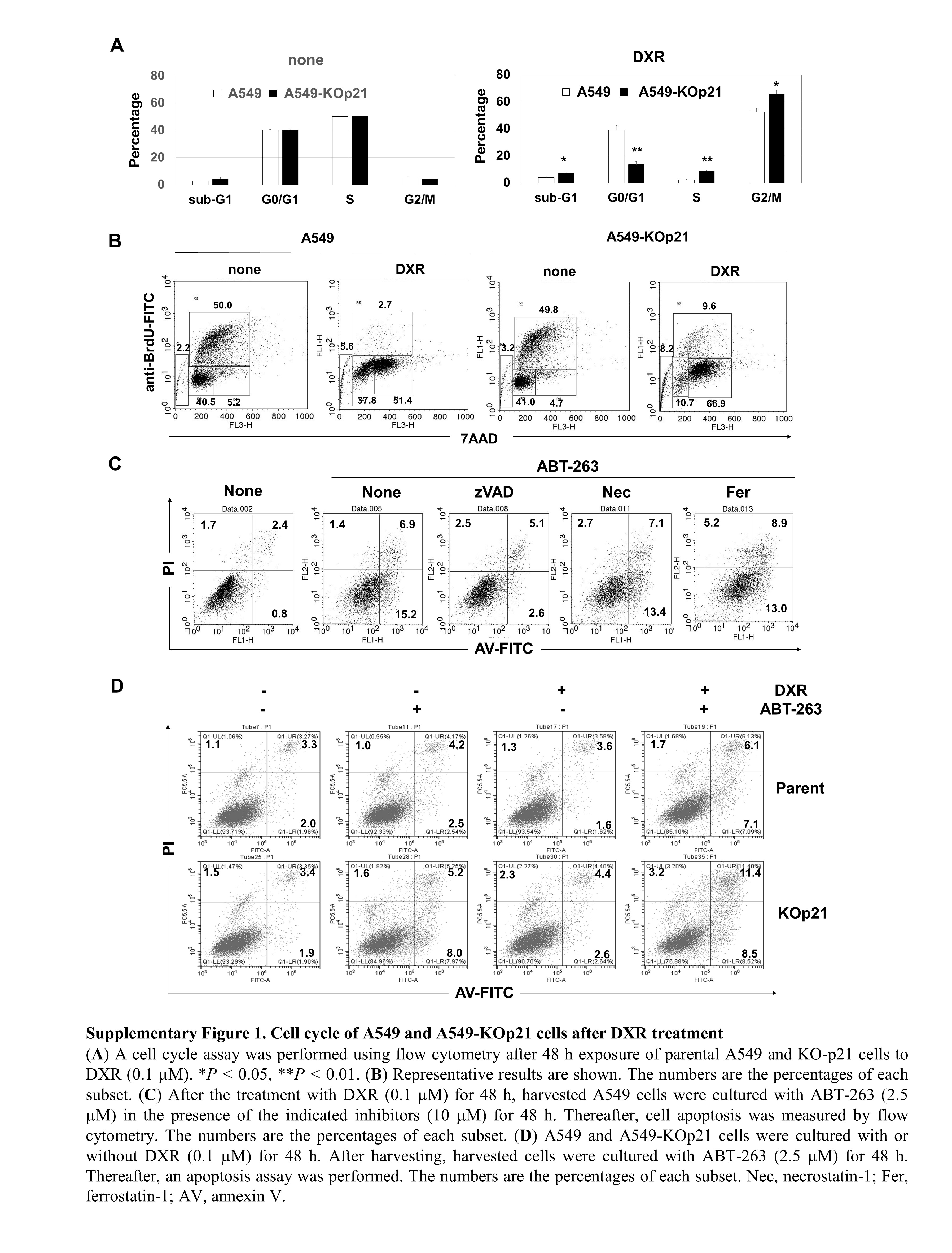

Supplement: Supplementary file 1 — FIGURE S1 Cell cycle of A549 and A549‐KOp21 cells after DXR treatment [file CPR-55-e13326-s002.JPG]

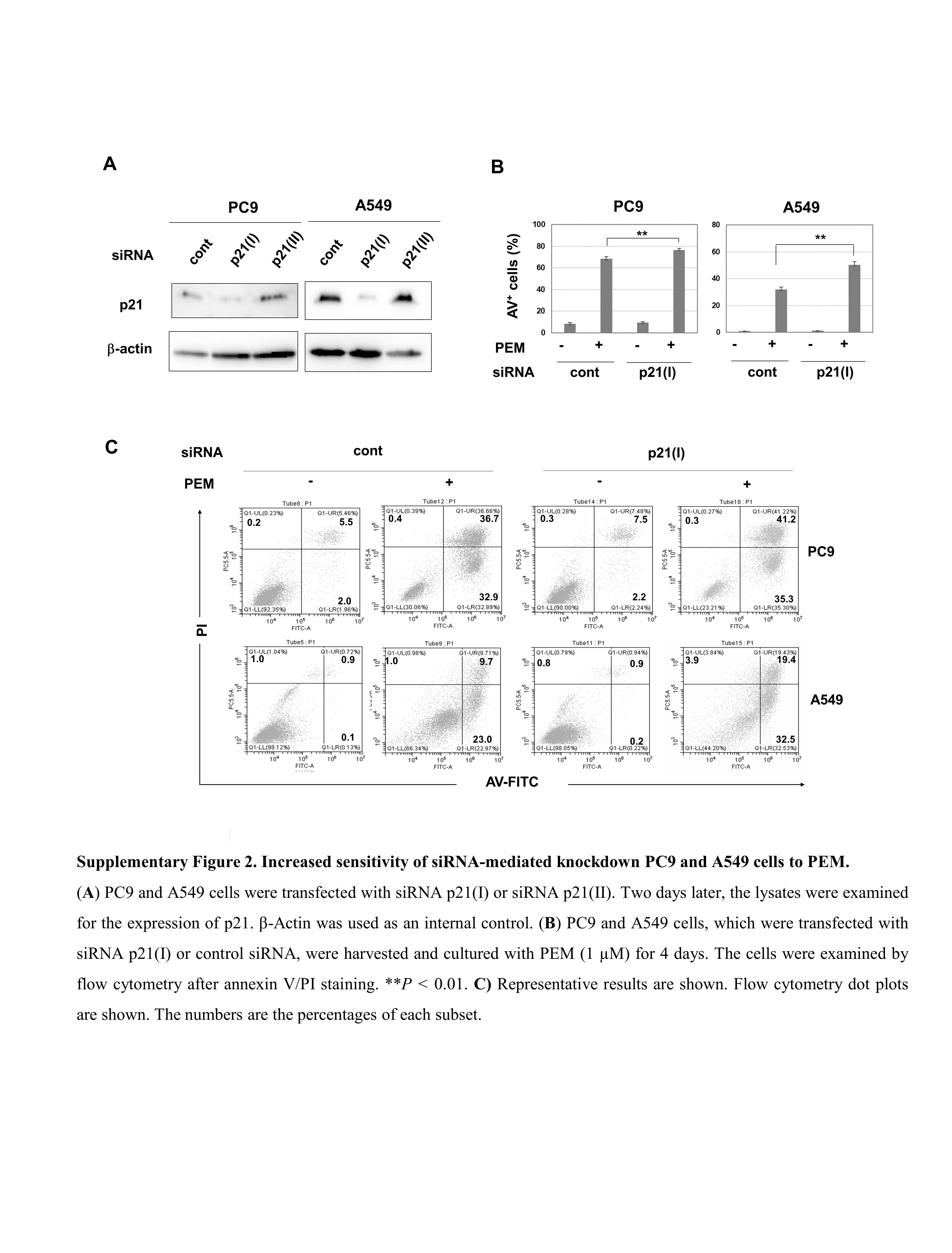

Supplement: Supplementary file 2 — FIGURE S2 Increased sensitivity of siRNA‐mediated knockdown PC9 and A549 cells to PEM [file CPR-55-e13326-s001.JPG]

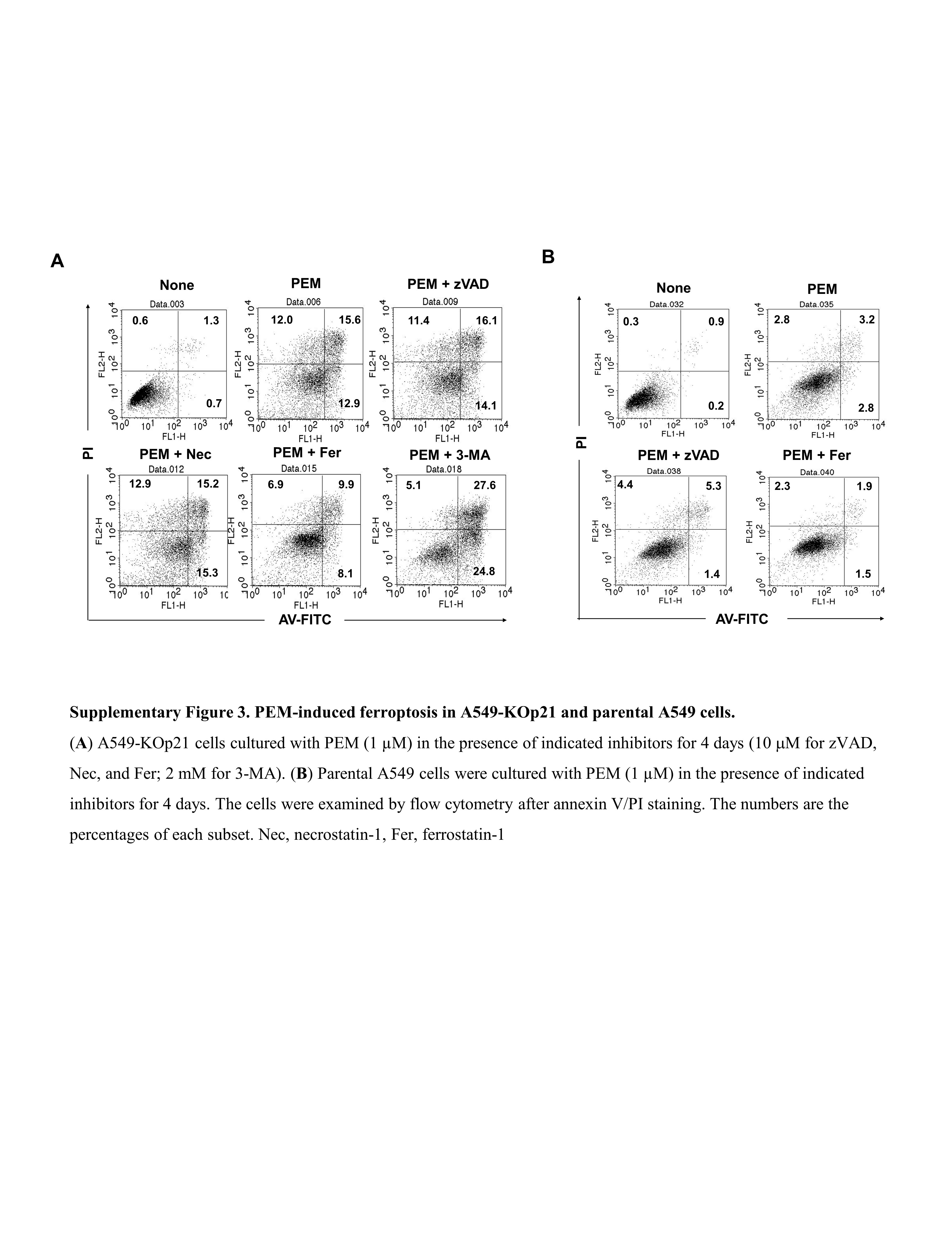

Supplement: Supplementary file 3 — FIGURE S3 PEM‐induced ferroptosis in A549‐KOp21 and parental A549 cells [file CPR-55-e13326-s003.JPG]
